# *ETV6/RUNX1*-positive childhood acute lymphoblastic leukemia in China: excellent prognosis with improved BFM protocol

**DOI:** 10.1186/s13052-018-0541-6

**Published:** 2018-08-16

**Authors:** Yu Wang, Hui-min Zeng, Le-ping Zhang

**Affiliations:** Department of Pediatrics, Peking University People’s Hospital, No.11 Xizhimen South Street, Beijing, 100044 China

**Keywords:** Acute lymphoblastic leukemia, *ETV6/RUNX1*, MRD, Prognosis

## Abstract

**Background:**

In childhood B-precursor acute lymphoblastic leukemia (B-ALL), the *ETV6/RUNX1* fusion transcript is considered to have an excellent outcome. However, few studies of children with *ETV6/RUNX1*-positive ALL from China have been conducted. It is largely unknown whether clinical outcomes for patients with this genotype and important factors that influence such outcomes are similar to those reported in other countries. Therefore, it is important to analyze the outcomes of children with *ETV6/RUNX1*-positive ALL treated at our institution with the aim of identifying significant prognostic variables in a Chinese population.

**Methods:**

We studied the clinical characteristics and treatment outcomes for 77 pediatric patients diagnosed with *ETV6/RUNX1*-positive ALL between 2005 and 2015 at our institution.

**Results:**

The 5-year event-free survival (EFS) and the disease-free survival (DFS) were reported to be 90% ± 3% and 96% ± 3% respectively. Two patients had a relapse at a median of 42 months from diagnosis and the 5-year cumulative incidence of relapse was 2.1%. Despite intensive chemotherapy or allogeneic hematopoietic cell transplantation, the 2 relapsed patients succumbed to the disease progression and the 5-year overall survival (OS) was 97% ± 2%. Multivariate analysis for EFS revealed that the minimal residual disease (MRD) ≥10^− 3^ on Day + 33 negatively affected the outcome.

**Conclusions:**

In conclusion, patients with *ETV6/RUNX1* fusion transcript can achieve a high rate of complete remission and the long-term curative effect was excellent under risk-stratified treatment. In case of relapse, the MRD level at the end of induction therapy should be taken into consideration while deciding the appropriate chemotherapy dosage.

## Background

Acute lymphoblastic leukemia (ALL) can be exactly classified according to immunophenotype and molecular aberrations since several somatic genetic alterations have been characterized during the last decades [1, 2]. In pediatric B-ALL, t (12; 21) (p13; q23) is the most common chromosomal abnormality [3]. It results from the juxtaposition of the transcription factor *ETV6* gene on chromosome 12 to the *RUNX1* sequences on chromosome 21 [3–6]. *ETV6/RUNX1*-positive ALL is considered to arise prenatally and may precede a pre-leukemic phase [7]. Furthermore, the presence of this fusion transcript alters the differentiation process and enhances self-renewal of hematopoietic progenitor cells, particularly of the B-lineage [8]. Based on the favorable molecular response to treatment and excellent clinical outcomes, it is believed that this rearrangement has prominent therapeutic significance [9, 10], although some recent long-term results have revealed late relapses among patients with *ETV6/RUNX1*-positive ALL [11, 12].

Recently, genome-wide analysis and whole-exome sequencing demonstrated that every *ETV6/RUNX1*-positive ALL patient could have multiple mutations, underscoring the heterogeneity of this subgroup [13, 14]. Moreover, this genetic heterogeneity may influence overall treatment response and survival. In this evaluation, we reviewed the outcomes of children with *ETV6/RUNX1*-positive ALL treated at our institution and attempted to identify significant prognostic factors with regards to survival.

## Methods

### Patients and treatment

Seventy seven patients diagnosed with *ETV6/RUNX1*-positive ALL from January 2005 to December 2015 at the Department of Pediatrics were included in the initial study cohort. The diagnosis and classification of ALL were based on immunophenotyping, cytogenetics, and molecular abnormality. The diagnosis of *ETV6/RUNX1* rearrangement was established by real-time quantitative polymerase chain reaction (RQ-PCR), as the detection rate of this translocation using conventional karyotyping (G-banding) analysis is less than 0.05% [15]. A bone marrow study was conducted every 2–3 months for MRD detection using RQ-PCR during the first 3 years from diagnosis. Thereafter, they were detected every half year for a total of 5 years.

**Fig. 1:**
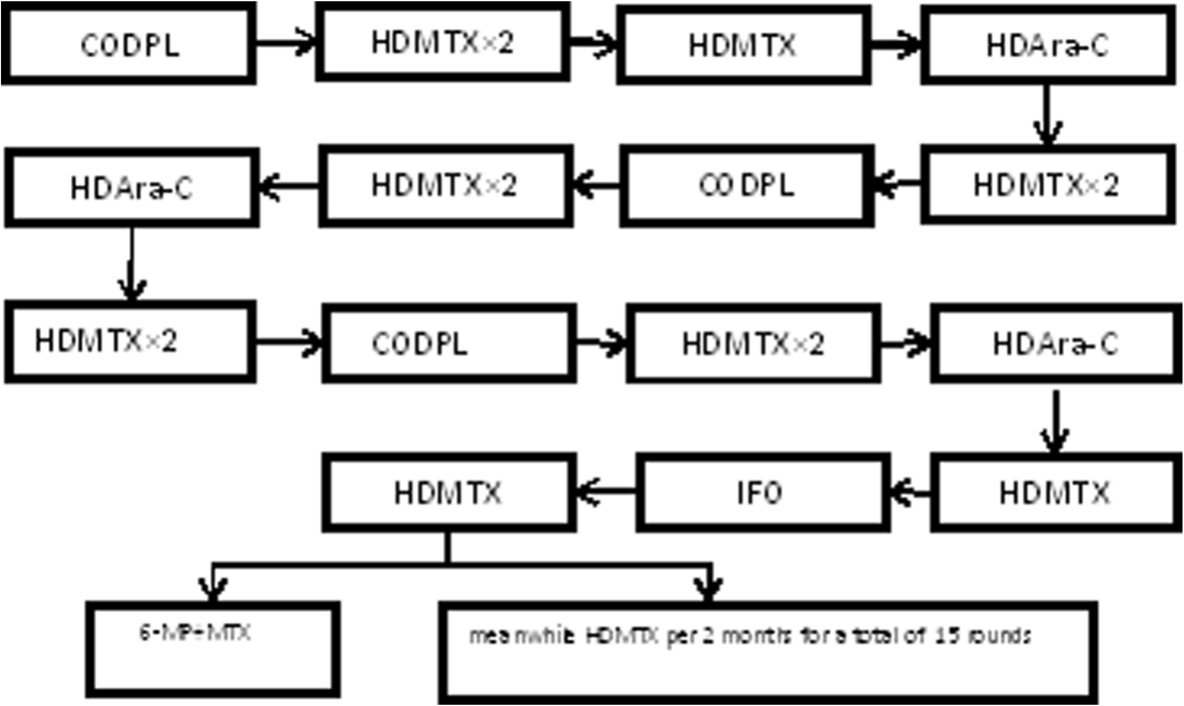
Flow chart of the improved BFM protocol at our institution. It includes remission induction, consolidation treatment, and maintenance therapy according to risk group

All the patients were treated according to the ALL-B protocol deriving from the improved Berlin-Frankfurt-Münster (BFM) protocol (Table 1 and Fig. 1). In brief, patients received a five-drug remission induction followed by the consolidation treatment and maintenance therapy. During the consolidation treatment, there were two courses of 4-week re-induction therapy. Besides, patients also received triple intrathecal therapy (TIT) treatments for a total of 20–22 doses to avoid central nervous system leukemia (CNSL). The whole treatment course lasts 3 years.

**Table 1:** Improved BFM protocols at our institution

Treatment and medicine	Dose	Time
Induction and re-induction therapy		
CODPL		
DEX or Pred	10 mg/m^2^(maximum:10 mg) or 60 mg/m^2^(maximum:60 mg)	d1–28(reduction in 1 week)
VCR	1.5 mg/m^2^(maximum:2 mg)	d1, 8, 15, 22
CTX	1 g/m^2^	d1
DNR or IDR	40-60 mg/m^2^ or 8-10 mg/m^2^	d1, 8
L-asp	10,000 U/m^2^	d15, 17, 19, 21, 23, 25, 27, 29, 31, 33
Consolidation therapy		
HDMTX×2		
HDMTX	2.5–3.5 g/m^2^	d1, 22
VCR	1.5 mg/m^2^	d1, 8, 15, 22
DNR or IDR	40-60 mg/m^2^ or 8-10 mg/m^2^	d8, 10
HDMTX		
HDMTX	2.5–3.5 g/m^2^	d1
VCR	1.5 mg/m^2^	d1
HDAra-C		
HDAra-C	2 g/m^2^	d1–3
DNR or IDR	40-60 mg/m^2^ or 8-10 mg/m^2^	d2–3
IFO		
IFO	1 g/m^2^	d1–5
VP-16	100 mg/m^2^	d3–5
VCR	1.5 mg/m^2^	d1
Maintenance therapy		
6-MP	50 mg/m^2^	once a day
MTX	20 mg/m^2^	once a week

*DEX* dexamethasone, *Pred* prednisone, *VCR* vincristine, *CTX* cyclophosphamide, *DNR* daunomycin, *IDR* idarubicin, *L-asp* native *Escherichia coli* L- asparaginase, *HDMTX* high dose methotrexate, *HDAra-C* high dose cytarabine, *IFO* ifosfamide, *VP-16* etoposide, *6-MP* mercaptopurine

Risk stratification was further refined by including MRD measurements at the end of remission induction therapy (cut-off, 10^− 3^) regardless of age and leukocyte count. The dose and intensity of anthracyclines as well as high-dose methotrexate differed by risk group.

Informed consent was obtained from the parent or guardian and assent obtained from the patient when appropriate.

### Statistical analysis

Overall survival (OS) was measured from the time of diagnosis to the date of last follow-up or death from any cause. Disease-free survival (DFS) was calculated from complete remission (CR) to the first hematologic relapse. Finally, event-free survival (EFS) was defined as the time from diagnosis of ALL to the last follow-up in CR or the first event that included a molecular relapse (recurrence of MRD), secondary malignancy, or death. Clinical analysis and comparison was estimated according to the method of descriptive analysis and Chi-square test. Kaplan-Meier survival curve and Log-rank test was used for survival analysis; *COX* regression analysis was performed in multivariate analysis. A statistically significant difference was set to a *p* value < 0.05.

## Results

### Patient characteristics

Some of the clinical characteristics are presented in Table 2. According to the immunophenotype, all 77 patients were diagnosed as B-precursor ALL. No t (12;21) expression was detected in 66 patients via conventional G-banding analysis. Cytogenetic abnormalities other than the cryptic *ETV6/RUNX1* rearrangement were identified by G-banding analysis. The most frequent structural aberrations analyzed by G-banding included those in chromosomes 12,6,21 and 9. Structural abnormalities involving chromosomes 12 and 6 were found in 7 and 9 patients, respectively and further characterized with addition of 12q and deletion of 6q. Trisomy for chromosomes 21 was detected in three patients, while loss of chromosome 21 was detected in another 3 patients.

**Table 2:** Clinical characteristics of patients with initial *ETV6-RUNX1*-positive ALL

Clinical features	number (ratio)	range	median
Gender			
male	48(62.3%)		
female	29(37.7%)		
Age (years)			4
<1	0		
1~ 6	59(76.6%)		
6~ 10	12(15.6%)		
>10	6(7.8%)		
WBC(×10^9^/L)			11
<20	54(70.1%)	0.4~ 20	5.3
20~ 50	12(15.6%)	21~ 48	33
>50	11(14.3%)	55~ 145	75
PLT(×10^9^/L)			48
<100	52(67.5%)	3~ 95	36.5
≥ 100	25(32.5%)	102~ 457	150
Immunophenotype			
Common-B	60(77.9%)		
Pre-B	17(22.1%)		
Chromosome	66(85.7%)		
hyperdiploidy	7(10.6%)		
hypodiploidy	6(9.1%)		
normal karyotype	45(68.2%)		
pseudodiploidy	22(33.3%)		
*ETV6-RUNX1/ABL* (%)	77(100%)	20.9~ 789.1	240.78
MRD on Day + 33(%)	71(92.2%)		
≥ 10^−2^	6(8.5%)	1.7~ 14.5	2.25
10^−2^~ 10^−3^	7(9.9%)	0.1~ 0.67	0.2
10^−3^~ 10^−4^	14(19.7%)	0.019~ 0.091	0.03
10^−4^~ 10^−5^	2(2.8%)	0.0049~ 0.0062	0.0055
negative	42(59.2%)	0	0

*WBC* white blood count, *PLT* platelets, *MRD* minimal residual disease detected by RQ-PCR

### Early treatment response

All 77 patients achieved CR at the end of remission induction chemotherapy (Day + 33). However, the level of MRD was not completely negative as analyzed by RQ-PCR techniques. As a result, 29 MRD-positive cases (*n* = 71, 40.8%) were detected ranging from 0.0049%~ 14.5% using RQ-PCR.

### Late treatment response

**Fig. 2:**
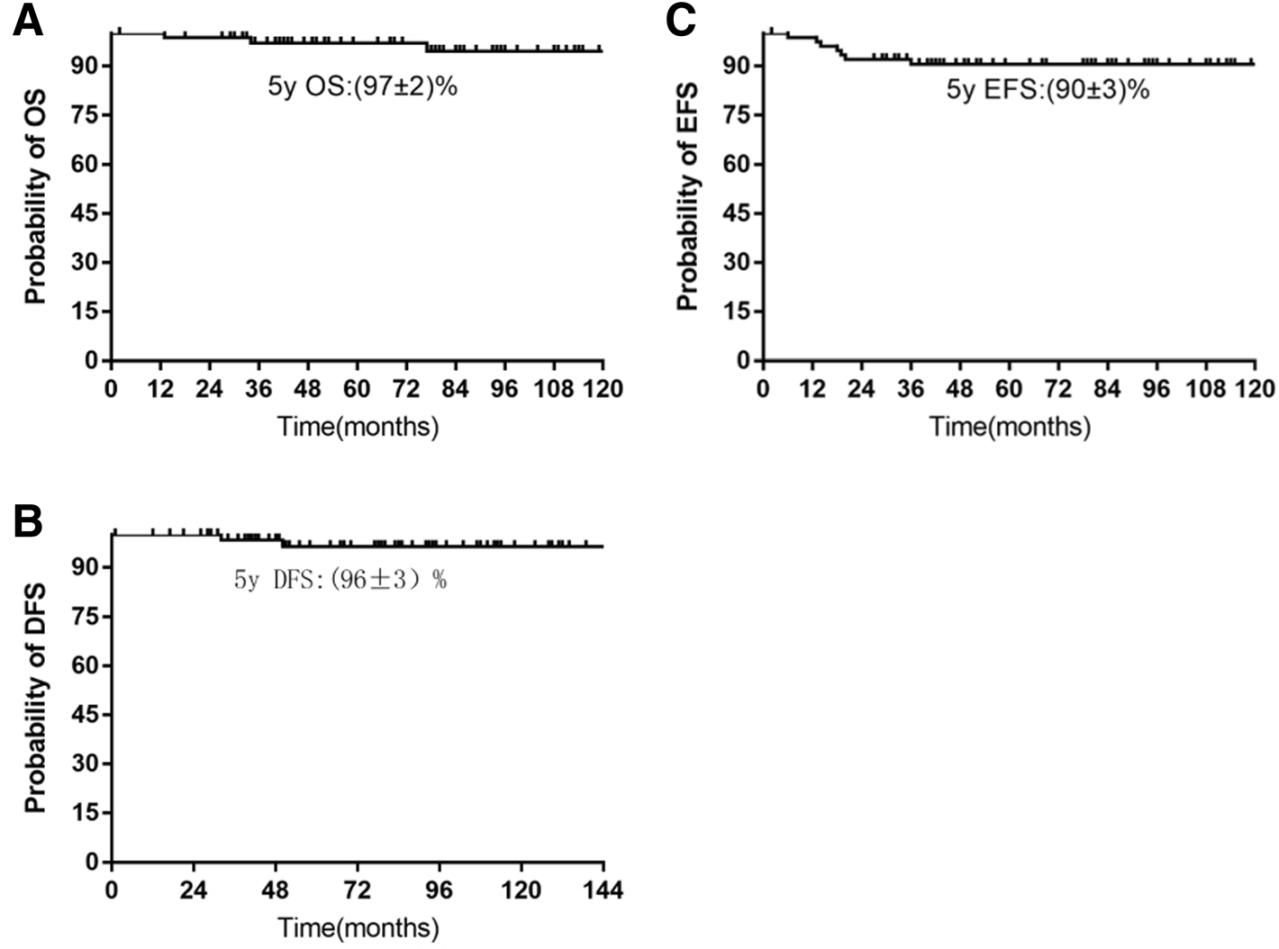
Kaplan-Meier estimates of OS (**a**), DFS (**b**) and EFS (**c**) in *ETV6/RUNX1* pediatric ALL patients by study. Rates at 5 years are reported as means ± standard errors. The probability of 5-year OS, DFS and EFS were reported to be (97 ± 2) %, (96 ± 3) %, and (90 ± 3) %, respectively

The median follow-up for this subgroup was 71 months (range 19–154 months). The probability of 5-year OS, DFS and EFS were reported to be 97 ± 2%, 96 ± 3%, and 90 ± 3%, respectively (Fig. 2). Two patients subsequently developed hematological relapse (presence of leukemic blasts > 5% in BM) at a median of 42 months from diagnosis (33 and 51 months, respectively). A 5-year cumulative incidence of relapse was calculated as 2.1%.

### Survival analysis

Univariate analysis of prognostic factors for EFS revealed that WBC > 50 × 10^9^/L and the MRD ≥10^− 3^ on Day + 33 had a significant impact, with the presence of other factors predicting worse outcome (Table 3). Multivariate analysis revealed that only MRD ≥10^− 3^ on Day + 33 was an independent risk factor for EFS (Table 4).

**Table 3:** Univariate analyses of factors influencing EFS

Factor	Patient	Event	*P*
Age (years)			0.351
< 5	51	3	
> 5	26	3	
WBC(×10^9^/L)			0.045
< 50	66	2	
> 50	11	4	
MRD on Day + 33			0.025
< 10^−3^	59	1	
≥ 10^−3^	13	5	
MRD on Day + 90			0.864
negative	66	5	
positive	11	1

**Table 4:** Multivariate analyses of factors influencing EFS

Factor	Hazard ratio (95% *CI*)	*P*
MRD on Day + 33		0.034
< 10^−3^	1	
≥ 10^−3^	2.416(1.480~ 12.169)	
WBC(×10^9^/L)		0.285
> 50	1	
< 50	0.173(0.034~ 0.877)	

*CI* confidence interval

## Discussion

Here, we report the clinical features, response to chemotherapy, prognostic factors and outcome for *ETV6/RUNX1-*positive ALL in Chinese children treated according to the improved BFM protocol at our institution.

Among variables that influenced EFS, we found MRD as detected by RQ-PCR for the *ETV6/RUNX1* transcript on Day + 33 as well as WBC > 50 × 10^9^/L at the time of diagnosis to be significant. Although leukocyte count, but not age, was found to be a prognostic factor in our study, many reports have suggested that National Cancer Institute (NCI)/Rome risk group status based on age and initial WBC count should be considered when treating *ETV6/RUNX1*-positive patients [16, 17]. However, according to our multivariate analysis only MRD on Day + 33 had a significant negative impact on EFS. Multiple available data has been established to determine the MRD level at the end of induction therapy in order to evaluate the response to chemotherapy [18]. In our cohort, the cut-off level of MRD at the end of induction therapy was 10^− 3^. In order to determine if a lower MRD level at such a time better defines poor prognosis, a greater number of patients need to be analyzed. In contrast to the prognostic relevance of MRD on Day + 33, lower level MRD on Day + 90 among fewer positive patients failed to show significance consistent with previous studies [18, 19].

According to our results, the treatment outcomes for this genetic subtype at our institution were excellent with OS and DFS rates greater than 90%, though 40% of the patients had detectable MRD at the end of induction therapy. The favorable outcome may be attributed to the assessment of risk stratification and differences in the intensities of chemotherapy in our treatment protocol. At our institution, patients with higher MRD levels were treated with more intensive chemotherapy regimens (relatively high dose shown in Table 1), together with a reduced treatment interval to eliminate the negative prognostic impact. Many studies have proven this opinion. For instance, DFCI ALL Consortium protocols before 1995 used a more rigorous criteria at lower risk [6, 20]. In fact, the outcome was superior and findings from other studies have supported this result indirectly [3, 6, 21]. Besides, previous studies have demonstrated that *ETV6/RUNX1*-positive lymphoblasts were exquisitely sensitive to high-dose methotrexate and L-asparaginase in vitro [22, 23]. Patients with *ETV6/RUNX1* fusion transcript may benefit from the intensive L-asparaginase and high-dose methotrexate employed in our protocol. A longer treatment course may also be one of the most important factors in determining prognosis, as the majority of relapses occur off-therapy [11], and even after 10–20 years [24]. These observations were consistent with Total Therapy study XV at St Jude Children’s Research Hospital [10]. Moreover, it is worth mentioning that there were no serious toxic complications, secondary malignancies and treatment-related mortality in our cohorts. But a longer follow-up time is needed to draw the correct conclusion.

Unfortunately, we were unable to explore *IgH/TCR* rearrangements or sequence the *ETV6/RUNX1* genome to have a better understanding of the two treatment failures.

In conclusion, our studies clearly indicate an excellent prognosis for *ETV6/RUNX1-*positive ALL patients. However, larger, prospective clinical trials will be needed to confirm whether *ETV6/RUNX1* can be a completely independent prognostic factor.

## Conclusions

In summary, our *ETV6/RUNX1*-positive ALL cohort had an excellent prognosis. Significant MRD at the end of remission induction chemotherapy adversely affected EFS. Larger scale studies will be necessary to confirm the factors that characterize a potential subset with poor outcome, and to improve the survival of this subtype.

## Abbreviations

ALL: Acute lymphoblastic leukemia
CNSL: Central nervous system leukemia
CR: Complete remission
DFS: Disease-free survival
EFS: Event-free survival
MRD: Minimal residual disease
OS: Overall survival
RQ-PCR: Real-time quantitative polymerase chain reaction
